# Stigma: The Stealth Weapon of the NTD

**DOI:** 10.1371/journal.pntd.0000230

**Published:** 2008-04-30

**Authors:** Peter J. Hotez

**Affiliations:** Department of Microbiology, Immunology, and Tropical Medicine, The George Washington University and Sabin Vaccine Institute, Washington, D.C., United States of America

The neglected tropical diseases (NTDs) are the most common infections of poor people in developing countries, where they cause a high disease burden that rivals HIV/AIDS, tuberculosis, or malaria [1]. The NTDs also exhibit important poverty-promoting features, a consequence of their ability to adversely affect child development, pregnancy outcome, and worker productivity [1],[2]. Over the last two decades or more, several important quantitative indicators have been used to measure these health and economic consequences. Employment of the disability-adjusted life year (DALY) has facilitated a comparison of NTD disease burden with better known conditions, while several estimates of the economic impact of selected NTDs, including hookworm infection, lymphatic filariasis, and trachoma, have provided insights on how these conditions prevent the poorest people in developing countries from escaping poverty [2].

There is also a third component to the NTDs that may be just as important as the health and economic effects of these diseases, but it is one that so far has been the least tangible and difficult to measure. I am referring to the horrific social stigma associated with many of the NTDs, particularly highly disfiguring diseases such as Buruli ulcer, leprosy, lymphatic filariasis, and onchocerciasis. The link between stigma and the NTDs go back to our earliest recorded history [3]. The medical detective and writer Berton Roueche observed that in addition to multiple biblical references to “unclean” people with leprosy, an ancient Egyptian pharoah was known to banish people with leprosy to edges of the Saharan Desert. He coined the term *lepraphobia* to describe how, at the height of the leprosy epidemic in Europe in the 12th to14th century, affected individuals were often subjected to their own mock funeral prior to banishment from their families and communities [4]. In some cases, they endured torture and execution [4].

Our concepts and definitions of what exactly stigma means have changed over time. In his landmark treatise entitled *Stigma: Notes on the Management of Spoiled Identity*, the social scientist Erving Goffman noted that the original use of the term came from the Greek and referred to a scar made with a pointed instrument, usually signifying an inferior social or moral status, such as being a criminal, traitor, or slave [5]. In early Christian times the use of the term was broadened to indicate a mark of disgrace or physical disorder, presumably with lepraphobia in mind, and later to a more modern definition that linked stigma to disqualification from social acceptance, either for physical or social reasons [5],[6]. Professor Mitchell Weiss at the Swiss Tropical Institute now defines health-related stigma as “a social process or related personal experience characterized by exclusion, rejection, blame, or devaluation that results from experience or reasonable anticipation of an adverse social judgment about a person or group identified with a particular problem” [7]. Further, “the judgment is medically unwarranted with respect to the health problem itself, just as stigma targeting other aspects of group identity is also unwarranted . . . ” [7].

Over the last decade, several key papers have emerged that illustrate how the stigma resulting from specific NTDs contributes substantially to disease burden and even poverty [8]–[11]. A common mechanism is the exacerbation of disease and suffering that result from significant delays in seeking medical attention. For instance, Jorge Alvar and his colleagues at the World Health Organization and the US Centers for Disease Control and Prevention recently pointed out that women have a higher disease burden from leishmaniasis than men because of reduced health care access, and because of their heightened social isolation from the disfigurement caused by the cutaneous form of the disease, which can prevent a young woman from being permitted to touch her children, enter into marriage, or remain married [3],[9]. Similarly, a team from Groningen University Hospital in the Netherlands has eloquently described how the disfiguring wounds of Buruli ulcer in Africa cause affected individuals to attempt to hide their disease because of the belief that it results from witchcraft or the “evil eye,” and as a result such individuals seek neither medical attention nor employment [3],[9]. In a previous issue of *PLoS Neglected Tropical Diseases*, we published a revealing study by Myrtle Perera and her colleagues, who noted how the stigma resulting from the disability and disfigurement of lymphatic filariasis causes affected individuals to avoid free government clinics leading to worsened illness, reduced career aspirations, and ultimately, an inexorable downward spiral to poverty [11].

In an upcoming issue of *PLoS Neglected Tropical Diseases*, Professor Weiss provides a fresh look at stigma and the social burden of NTDs by looking at some of the disease-specific elements of stigma, including cultural meanings for infections such as leprosy, or how some of the more common disfiguring NTDs such as Buruli ulcer, leishmaniasis, lymphatic filariasis, and onchocerciasis produce stigmata primarily from physical features [12]. He further suggests a new framework for looking at how the known elements of stigma that results from these infections might provide a basis for establishing an effective health policy that is just as attentive to the social and cultural issues as they are the biological and medical ones [12]. To my mind, Professor Weiss makes the case that addressing these social underpinnings of the NTDs could be almost as important for achieving Millennium Development Goals and sustainable poverty reduction as mass drug administration and the development of new vaccines and other control tools [1]. I am not alone. In his previous role as Director of the Fogarty International Center of the US National Institutes of Health, Professor Gerald Keusch (now Deputy Provost for Global Health at Boston University) launched an innovative program to fund proposals on the role of stigma in health and disease. The Fogarty initiative recognizes that the social burden of disease occurs disproportionately in developing countries, that the type and degree of stigma vary across the disease condition, the country, and the culture, and that there is not a “one size fits all” interdisciplinary approach to reduce stigma [13],[14]. Of particular importance, these factors can potentially be addressed by high-quality social science research and evidence-based approaches [13],[14].

An important stimulus for establishing *PLoS Neglected Tropical Diseases* was the recognition that these conditions, possibly more than any other, are linked to social factors that rank in importance with the biological factors of the parasites and their hosts, the clinical features, and the large-scale approaches that rely on preventive chemotherapy and vector control. All of these factors relate to the concept of reducing the suffering from NTDs as a fundamental human right [1],[15]. We therefore continue to strongly encourage papers with solid, evidence-based social research as it applies to the NTDs.
